# Stakeholder perspectives from 15 countries in Africa on barriers in snakebite envenoming research and the potential role of research hubs

**DOI:** 10.1371/journal.pntd.0011838

**Published:** 2023-12-13

**Authors:** Ymkje Stienstra, Leslie Mawuli Aglanu, Janna M. Schurer, Rhona Mijumbi, Jean Bosco Mbonigaba, Abdulrazaq G. Habib, Brent Thomas, Jonathan Steinhorst, Rachael Thomson, Sara Padidar, John H. Amuasi, George O. Oluoch, David G. Lalloo

**Affiliations:** 1 Centre for Snakebite Research and Interventions, Liverpool School of Tropical Medicine, Liverpool, United Kingdom; 2 University of Groningen, Department of Internal Medicine/Infectious Diseases, University Medical Centre Groningen, Groningen, The Netherlands; 3 Global Health and Infectious Diseases Research Group, Kumasi Centre for Collaborative Research in Tropical Medicine, Kumasi, Ghana; 4 Bernhard Nocht Institute of Tropical Medicine, Hamburg, Germany; 5 Center for One Health, University of Global Health Equity, Butaro, Rwanda; 6 Department of Global Health and Infectious Disease, Cummings School of Veterinary Medicine, North Grafton, United States of America; 7 Malawi-Liverpool-Wellcome Programme, Liverpool School of Tropical Medicine, Liverpool, United Kingdom; 8 Rwanda Neglected Tropical Diseases Programme, Rwanda Biomedical Centre, Ministry of Health Kigali, Rwanda; 9 Bayero University Kano, Kano state, Nigeria; 10 Department of Clinical Sciences, Liverpool School of Tropical Medicine, Liverpool, United Kingdom; 11 Eswatini Snakebite Research and Intervention Centre, Simunye, Eswatini; 12 Eswatini Antivenom Foundation, Simunye, Eswatini; 13 Department of Biological Sciences, University of Eswatini, Kwaluseni, Eswatini; 14 Department of Global Health, School of Public Health, Kwame Nkrumah University of Science and Technology, Kumasi, Ghana; 15 Kenya Snakebite Research & Intervention Centre, Kenya Institute of Primate Research, Ministry of Health, Karen, Nairobi, Kenya; Rajarata University of Sri Lanka Faculty of Medicine and Allied Sciences, SRI LANKA

## Abstract

Snakebite envenoming is a debilitating neglected tropical disease disproportionately affecting the rural poor in low and middle-income countries in the tropics and sub-tropics. Critical questions and gaps in public health and policy need to be addressed if major progress is to be made towards reducing the negative impact of snakebite, particularly in the World Health Organisation (WHO) Africa region. We engaged key stakeholders to identify barriers to evidence-based snakebite decision making and to explore how development of research and policy hubs could help to overcome these barriers. We conducted an electronic survey among 73 stakeholders from ministries of health, health facilities, academia and non-governmental organizations from 15 countries in the WHO Africa region. The primary barriers to snakebite research and subsequent policy translation were limited funds, lack of relevant data, and lack of interest from policy makers. Adequate funding commitment, strong political will, building expert networks and a demand for scientific evidence were all considered potential factors that could facilitate snakebite research. Participants rated availability of antivenoms, research skills training and disease surveillance as key research priorities. All participants indicated interest in the development of research and policy hubs and 78% indicated their organization would be willing to actively participate. In conclusion, our survey affirms that relevant stakeholders in the field of snakebite perceive research and policy hubs as a promising development, which could help overcome the barriers to pursuing the WHO goals and targets for reducing the burden of snakebite.

## Author summary

Snakebite envenoming is a debilitating neglected tropical disease disproportionately affecting the rural poor in low and middle-income countries in the tropics and sub-tropics. Evidence gaps need to be addressed if major progress is to be made towards reducing the negative impact of snakebite, particularly in the World Health Organisation (WHO) Africa region. We conducted an electronic survey among 73 people working at ministries of health, health facilities, academia and non-governmental organizations from 15 countries in the WHO Africa region. They reported the primary barriers to snakebite research and policy making to be limited funds, lack of relevant data, and lack of interest from policy makers. Adequate funding commitment, strong political will, building expert networks and a demand for scientific evidence were all considered potential factors that could facilitate snakebite research. All participants indicated interest in the development of research and policy hubs.

## Introduction

To achieve the target of halving snakebite mortality and disability by 2030 [1], critical questions and gaps in public health and policy need to be addressed. Globally, it is estimated that up to 5.5 million snakebites occur annually, resulting in about 1.8–2.7 million envenoming and mortalities ranging between 81,000 and 138,000 [2,3]. In sub-Saharan Africa, the actual incidence of snakebite may vary from 100 to 650 bites per 100,000 inhabitants per year [4]. Snakebite is estimated to cause 1.03 million (95% Confidence Interval: 0.80–1.28 million DALYs) disability adjusted life years (DALYs) per year in sub-Saharan Africa [5]. This estimated DALYs is based on hospital reported data on snakebite envenoming (SBE) related deaths and amputations, and an extrapolation of the proportion of Post-Traumatic Stress Disorder (PTSD) from Asia. The scarcity of viable population-level data on snakebite and the variability of data capture approaches in various sub-Saharan Africa countries hinders efforts towards accurately estimating the regional disease burden [6].

The addition of snakebite envenoming into the Category A of the Neglected Tropical Diseases (NTDs) [7], the subsequent launch of the strategy for prevention and control [1] and its inclusion in the 2030 NTD Road Map [8], has attracted global research and investment interests. Despite commitment at the global level, there is significant concern about meeting the targets for burden reduction in Sub-Saharan Africa. Many clinical and public health challenges remain, including lack of affordable, safe and efficacious antivenoms in the region [1,9]. There also remains limited research evidence to support clinical and public health decision making, including epidemiology, economic burden, prevention, and therapeutic tools. Mismatched priorities between funders, researchers and policy-makers hinders the translation of health research evidence into practice. This is further exacerbated by poor communication mechanisms, lack of high-quality research and the lack of capacity for policy-makers and stakeholders to access and use the available health research [10–12].

Launched in November 2012, the World Health Organisation (WHO) introduced the Strategy on Health Policy and Systems Research to advocate for improvement in alignment between research evidence and decision making through close collaboration between researchers and decision-makers [13]. Further arguments have been made for more deliberate researcher-practitioner collaboration, including the development of knowledge translation platforms [14]. Strategic research hubs involving various stakeholders have been advocated to provide a platform for prioritised knowledge generation, while taking into account the complexity of health policy making [15,16].

Engaging researchers, health practitioners, policy makers and other stakeholders in setting the snakebite research agenda through priority alignment is essential to harness national and regional interests. In the Strategy for Prevention and Control of Snakebite Envenoming, research partnership and collaboration is identified as key to meeting the set targets [1]. However, successfully promoting evidence-based decision making for snakebite in Africa requires the existence of a receptive climate to lay the foundation for collaboration among stakeholders from diverse disciplines. The sustainability of translational processes from evidence into practice also requires the consideration of national and regional priorities, high level advocacy, technical capacity and a concerted action from all relevant stakeholders [17].

Against this backdrop, we engaged snakebite stakeholders from various disciplines in 15 WHO Africa region countries to identify barriers to snakebite evidence-based decision making at national and regional levels and to explore whether developing regional research hubs as a research and policy initiative could help to overcome these barriers.

## Methods

### Ethical statement

Ethical approval was granted by the Liverpool School of Tropical Medicine Research Ethics Committee (21–068), Liverpool, UK. All participants received an information sheet and provided written informed consent digitally. All data was stripped of identifiable information. Where needed, descriptions of participants were generalized to maintain confidentiality.

### Recruitment of participants

We used purposive sampling with snowballing to include snakebite envenoming stakeholders working in 15 countries in Africa: Benin, Burkina Faso, Burundi, Cameroon, Chad, Eswatini, Ethiopia, Ghana, Kenya, Nigeria, Rwanda, South Sudan, Tanzania, Togo, and Uganda. Participants were recruited between October 2021 and March 2022. We aimed to include four to five stakeholders per country. The initial stakeholders in each country were identified based on their active engagement in snakebite related activities in policy, community engagement, academia or research. The link to the survey was shared to them via email. The snowballing technique included requests for the survey to be shared amongst colleagues by the initially identified stakeholders and for the survey to also be posted on websites targeting policy makers. The exact number of stakeholders who read or received the invitation is therefore unknown. Invited participants were health care workers, scientists and policy makers involved in snakebite envenoming. Professionals working for international non-governmental organizations or universities, but not primarily based in one of these 15 countries, were excluded. The survey is attached as S1 Appendix.

### Electronic survey

An electronic survey was developed to explore participants’ views on the barriers and facilitators to snakebite research and policy making, the potential role of research hubs, and the topics to prioritize in these hubs. Questions were multiple-choice, open-ended or scored on a 5-point Likert scale (strongly disagree, disagree, neutral, agree, strongly agree). Questions on barriers, facilitators and priorities were based on discussions within the research team as well as those found in a scoping review of researchers’ involvement in health policy dialogue in Africa [18]. Participants were asked to rank the list of topics in order of priority from the first through to the twelfth. The survey was available both in English and in French and took approximately 30–45 minutes to finish based on the REDCap reports. The survey was translated to French by a native speaker active in public health research. The answers to the open-ended questions were translated to English by the study team.

### Data analysis

REDCap (Research Electronic Data Capture, version 11, Vanderbilt University) was used to record survey responses. Descriptive statistics (in SPSS version 27, IBM Corp., Armonk, NY) were used to analyse the survey data. Participants were coded based on their affiliation and the order in which their responses were received. The affiliation codes are H: health facility or university medical centre; MIN: Ministry of Natural Resources, other; MNTD: Ministry, NTD programme; MoH: Ministry of Health; MSB: Ministry of Health, Snakebite contact person; NGO: non-governmental organization; R: research facility or university. The order of priority of the predefined snakebite research hubs focus areas were based on participants’ individual rankings. The number of stakeholders who selected a particular topic as either first, second or third priority were summed and used to rank the overall level of priority. Stakeholders’ self-reported influence, interest and attitude was used to group stakeholders based on Eden and Ackerman’s classification as: *players* (medium or high influence and interest), *context-setters* (medium or high levels of influence but none or low levels of interest), *subjects* (none or low levels of influence but medium or high levels of interest) and *crowd* (none or low levels of influence and interest) [19,20]. Stakeholders’ attitudes regarding the initiative were included in the matrix using a colour for the participant codes (green-for, black-neutral and red-against).

## Results

### Participants

In total 73 stakeholders participated in the survey (Table 1). The participants worked for the ministries of health (n = 17, 23%), a ministry of natural resources (n = 1, 1%), health facilities or university medical centres (n = 18, 25%), research facilities or universities (n = 27, 37%), and non-governmental organizations (n = 10, 14%). Non-governmental organizations were active in wildlife conservation, improving access to antivenoms, promoting health and/or supporting health care systems.

**Table 1:** Number of stakeholders participating per country.

Country	Frequency	Percentage
Kenya	10	13.7
Ghana	8	11.0
Nigeria	7	9.6
Uganda	7	9.6
Cameroon	6	8.2
Eswatini	6	8.2
Tanzania	6	8.2
Ethiopia	5	6.8
Benin	4	5.5
Burkina Faso	3	4.1
Burundi	3	4.1
Rwanda	3	4.1
Togo	3	4.1
Chad	1	1.4
South Sudan	1	1.4

### Barriers and facilitators

**Fig 1:**
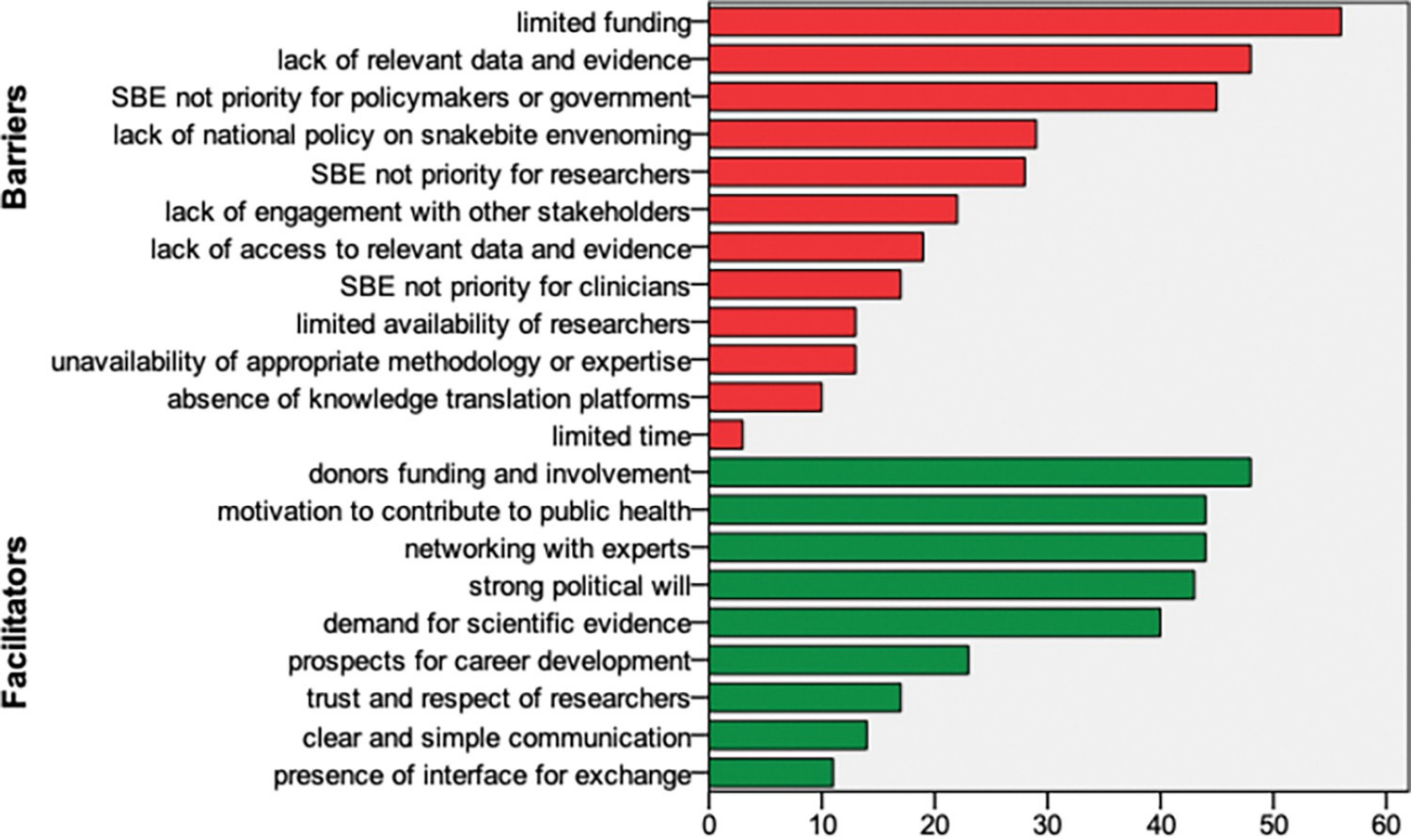
Frequency of items reported by stakeholders as barriers (in red) and facilitators (in green) in generating evidence and its translation into policy. Stakeholders could select max 5 items.

Sixty-six participants completed the questions on barriers and facilitators. Limited funding, lack of access to relevant data and evidence, and a low priority of SBE for policy makers or governments, were the most frequently mentioned barriers in evidence generation and translation into policy. There were no differences in the barriers reported by the stakeholders working for the Ministry of Health or Ministry of Natural Resources and the other stakeholders. The facilitators identified were donors funding and involvement, networking with experts, motivation to contribute to public health, strong political will, and demand for scientific evidence (Fig 1).

The open questions did not reveal any new topics apart from the barriers and facilitators included in Fig 1, but provided justifications for stakeholders’ choices.

‘*The lack of research (epidemiology/fundamental/operational) providing evidence which can be translated into public health messages*.*’* (R58, Director of a research institute)

‘*Previous official neglect but now remedied by reclassification of SBE under the NTD office*.’ (R10, lecturer at a university)

‘*Lack of incidence & prevalence data hampered strategic policy formulation*. *Snakebites are now reported and this results in increased studies that are triggering more multi-sector response to reverse the situation*. *This came also as an effect of consideration of SBE in the national NTD strategy*.’ (MNTD29, Ministry of Health, director of NTD program)

‘*1*. *Stakeholders platforms to share and express views and opinions*. *2*. *External influence from partners like WHO*. *3*. *Regional and global strategies*, *treaties and protocol*. *4*. *High interest from lead department at ministry of health*, *wildlife conservation organizations and Non-Governmental Organizations*.*’* (NGO16, working at wildlife conservation organization)

### Stakeholders’ priorities in research hubs

**Fig 2:**
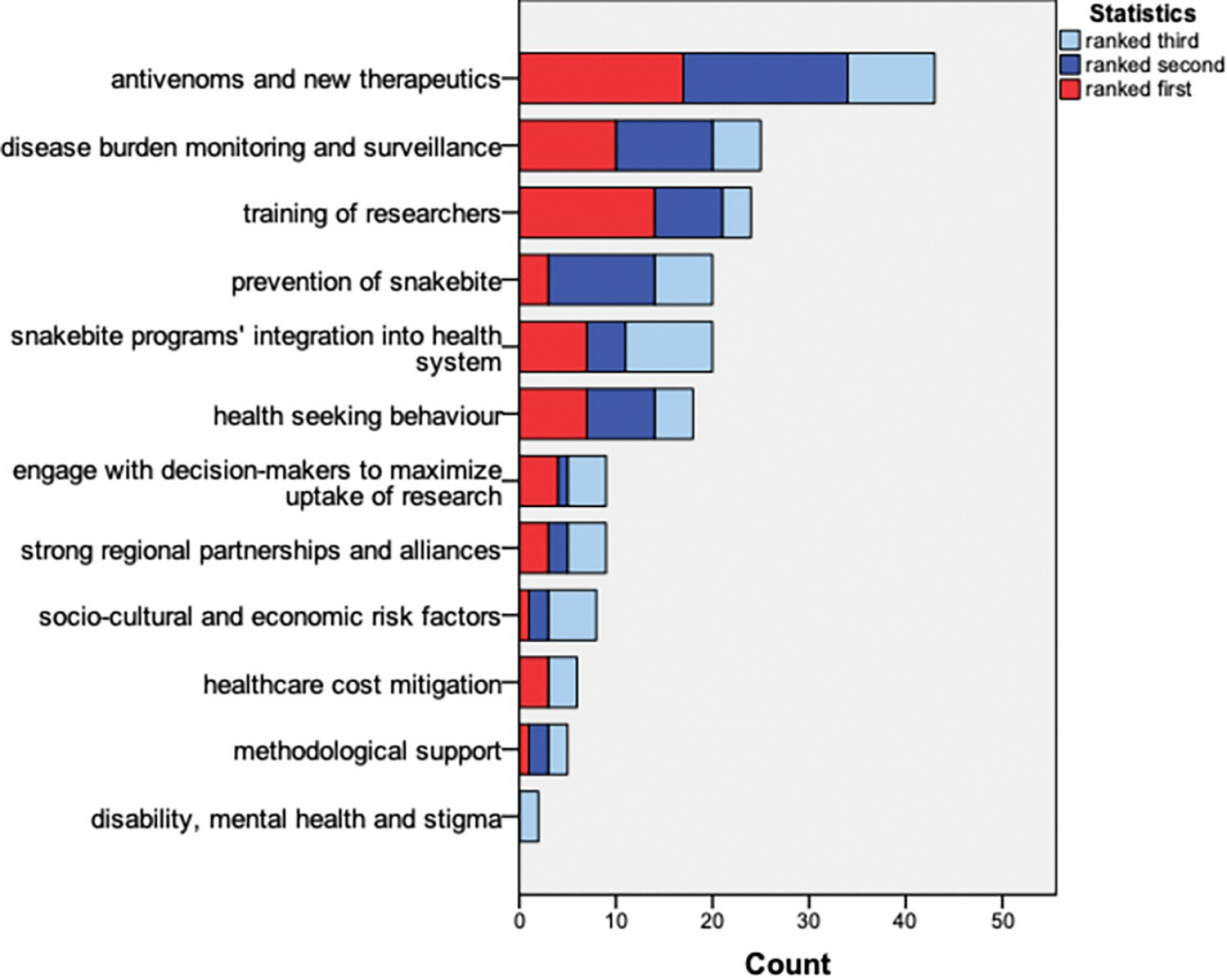
Stakeholders’ top three priorities for snakebite research hubs. The number of stakeholders who selected a particular topic as either first, second or third priority were summed and used to rank the overall level of priority.

The overall top three reported priorities for research hubs to address were (1) research on antivenoms and new therapies, (2) disease burden and surveillance, and (3) training researchers (Fig 2). These priorities differed between ministry-based stakeholders and others. The ministry-based stakeholders identified (1) antivenoms and new therapies, (2) disease burden and surveillance and (3) the snakebite programme integration into health systems as the top three priorities. Among the other stakeholders, the top three priorities reported were (1) antivenoms and new therapies, (2) training of researchers, (3) prevention of snakebites, and disease burden and surveillance (ranked third equally). The open questions did not reveal new topics apart from the twelve included in Fig 2, but provided justifications for stakeholders’ choices.

‘*Generate interest and networking among researchers*. *That would create evidence and would mobilise policy makers and resources*.’ (MNTD67, Ministry of health, NTD program)

‘*Low molecular weight snake bite treatments since the current first line treatments i*.*e*. *anti-snake venoms are plagued by the problems of anaphylaxis and consequent loss of acceptance as well problems of requiring specialized facilities/trained professionals for administration’* (R10, Lecturer involved in preparing national guidelines on SBE)

‘*We lack a coordinated system nationally*. *Some areas are very remote*, *that victims do find it difficult to reach medical facilities*. *Treatment of snakebite case is also extremely expensive*.*’* (R26, herpetologist active in increasing SBE awareness)

**Fig 3:**
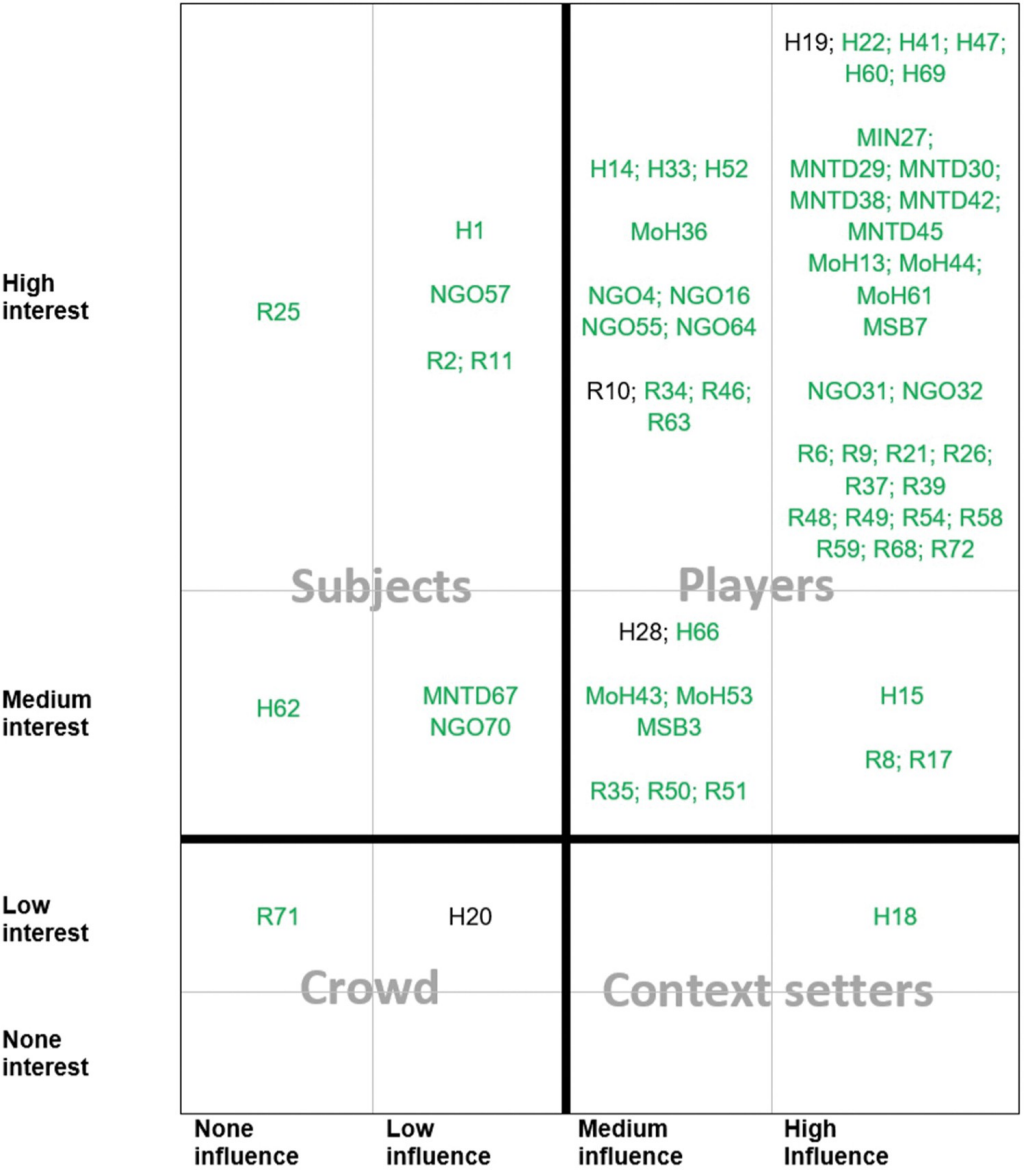
Influence, interest and attitude as self-reported by stakeholders towards research hubs. The colours in the figure indicate the attitude towards the research hubs: green (for) and black (neutral). Meaning of participants’ codes: **H**: health facility or university medical centre; **MIN**: Ministry of Natural Resources, other; **MNTD**: Ministry, NTD programme; **MoH**: Ministry of Health; **MSB**: Ministry of Health, Snakebite contact person; **NGO**: non-governmental organization; **R**: research facility or university.

The stakeholders included in the survey are mainly classified as players (83%, high interest combined with high influence) by the Eden-Ackermann’s classification (Fig 3). There were no stakeholders who had no interest in the research hubs. Of the 65 stakeholders included in the matrix, 51 (78%) held positions or were working at organizations mentioned as key stakeholder positions or organizations by other stakeholders from the same country.

Stakeholders were asked to which extent they thought their organization wanted to be involved in a research hub initiative. Fifty-one out of 65 (78%) indicated their organization would want to actively participate in the process including decision making. Four stakeholders out of the total group of stakeholders indicated their organization wanted to actively participate in the process without making decisions. Five stakeholders wanted to serve as a consultative body and one stakeholder only wanted to receive information on the initiative. Four could not answer this question on behalf of their organization in this stage.

### Stakeholder concerns

When asked for concerns about a potential initiative, stakeholders frequently mentioned financial means and sustainability. There were also concerns which relate to the collaboration and the organizational structure of the initiative as illustrated by the quotes below.

‘*Inclusiveness and equity’* (MSB3, Snakebite program at Ministry of Health)

‘*How will inter-country dialogue happen*?’ (NGO4, working at NGO promoting health in communities)

‘*Dominance in priorities as well policymaking/ decision making by well-funded foreign partners*.’ (R10, Lecturer at university)

‘*The main concern will be the inactivity or the poor motivation of authorities at the highest level*.’ (NGO64, research institute)

## Discussion

Limited funding, a lack of relevant data and evidence, and a perceived lack of priority amongst policy makers were reported by snakebite stakeholders from 15 countries in the WHO Africa region as top barriers to improving clinical decision making and public health policy. Apart from the availability of donor funding support, other facilitators emphasized by the stakeholders were the need for strong political will, stakeholders’ motivation to contribute to public health and building networks among experts with similar interest.

These findings support prevailing thoughts on barriers to evidence-based decision making in health [11,12,15,18,21]. Limited funding mechanisms was the most commonly highlighted barrier for SBE evidence generation and translation into policy. This comes as no surprise for a recently classified NTD. Although snakebite is gradually gaining support and investment momentum [9], the current funding and investment trend is not sufficient to sustain benefits for at-risk populations. National NTD programmes are challenged by the addition of a new NTD to their portfolio, often without an increase in an already limited budget. The lack of relevant data and evidence on the true burden of the disease was attributed to the poor financial commitment to snakebite research which was in turn associated with snakebite envenoming not being a priority for governments and policy-makers in LMICs. To increase public health interest and financial commitment, donor and government agencies require robust evidence of the disease burden to ignite the need for prioritisation and further push for a strong political will to facilitate political advocacy and drive research investment. These findings indicate a vicious cycle that perpetuates the neglect of snakebite envenoming research and control measures.

Creating a network of experts with similar interests was reported to be important in facilitating the process. This network will need a cross-disciplinary approach to tackle the priorities for research hubs reported by the stakeholders. The priorities reported were: safe and effective antivenoms and other new therapeutics; generation of evidence on the burden and impact through monitoring and surveillance; and training of researchers. Stakeholders from government ministries more often regarded disease burden and surveillance, and the snakebite programme integration into health systems as priorities. The cross-disciplinary approach should also include an early collaboration between policy makers and researchers, as such active involvement and collaboration has been identified as a motivator to using resulting evidence in policy practice [11,22]. A study from Malawi shows that between 2002 and 2017, health research evidence contributed minimally to the development of health policies and also had very little influence on decision making processes [22]. This was due to the weak capacity of stakeholders to access relevant data, low quality of available health research evidence and the non-involvement of health researchers and other stakeholders in the process. Health researchers and academics were involved in development of only 13% of the policy documents evaluated for the study. Evidence from such studies shows the need to raise and maintain commitment and political interest from policy-makers, particularly those from state ministries and other governmental agencies. Other studies have also highlighted the importance of training and capacity building for researchers and other stakeholders in enhancing both evidence generation and its dissemination in a manner that promotes its translation into policy actions [11,12,15,21,23].

Evidence from the Sub-Saharan Africa Network for TB/HIV Research Excellence (SANTHE) consortium provides a good example of how research hubs can contribute to the generation and translation of research knowledge into practice. The establishment of the SANTHE research consortium led to the empowerment of researchers, provided opportunities for quality training and an effective platform for information exchange and collaboration [24]. Similarly, a scientific hub based in South Africa uses its capacity to provide evidence-informed decision making support to the National Immunization Technical Advisory Groups (NITAG) in the WHO Africa Region [25]. In another example, members of the Developing Excellence in Leadership, Training and Science (DELTAS) Africa consortia highlighted access to funding, inclusive and engaging leadership, networking and diverse interface for interactions with experts as benefits gained from its establishment [26]. These factors are described to be influential in optimising the outcomes of the consortium. Other African initiatives that focus on the advancement of research and implementation of interventions in the field of NTDs include the African Research Network for NTDs and the Kikundi community of practice for NTD programme managers in Africa.

Establishing snakebite research hubs in Africa could leverage this potential to help achieve the WHO targets. The majority of the stakeholders included in the survey could be considered as players (high interest combined with high influence) by the Eden-Ackermann’s classification. All stakeholders expressed interest in an approach to fill the evidence and policy gaps and the majority indicated their organisation would want to actively participate in the process, including decision making. Limitations of the study include the unknown response rate and the low number of participants from Chad and South Sudan despite the considerable effort invested identifying potential participants.

Nevertheless, the interest of qualified, experienced, influential and active multidisciplinary stakeholders who participated in this study affirms that the establishment of snakebite envenoming research hubs would be a promising and welcome innovation.

## Conclusion

In order to make major progress in reducing the burden of snakebite, gaps in clinical decision making and public health policies need to be addressed. The lack of relevant data and evidence on the true burden of SBE have resulted in a vicious cycle that perpetuates the neglect of research and control measures. Our results suggest significant investment and commitment is needed to catapult and sustain snakebite research while strengthening institutional capacity to access and use the generated evidence in decision making. Snakebite research hubs, established equitably and with political support, could credibly synergise interest across stakeholders and ensure the right context-specific evidence is generated and made available for decision making.

## Supporting information

S1 Appendix Snakebite survey. (PDF)
