# Supplementary material for: Stakeholder perspectives from 15 countries in Africa on barriers in snakebite envenoming research and the potential role of research hubs
Source: PLoS Negl Trop Dis. 2023 Dec 13;17(12):e0011838. doi: 10.1371/journal.pntd.0011838 (PMC10752511; doi:10.1371/journal.pntd.0011838)
Supplement: S1 Appendix — (PDF) [file pntd.0011838.s001.pdf]

Snakebite

We would like to hear your thoughts on barriers in doing research on snakebite envenoming and your thoughts on the potential role of research hubs.

Section 1 (This is a section header with descriptive text. It only provides informational text and is used to divide the survey into sections for organization. If the survey is set to be displayed as "one section per page", then these section headers will begin each new page of the survey.)

Where are you based?

- ◯ Benin
- ◯ Burkina Faso
- ◯ Burundi
- ◯ Cameroon
- ◯ Chad
- ◯ Eswatini
- ◯ Ethiopia
- ◯ Ghana
- ◯ Kenya
- ◯ Nigeria
- ◯ Rwanda
- ◯ South Sudan
- ◯ Tanzania
- ◯ Togo
- ◯ Uganda
- ◯ other

Which? ________________________________

What is your position? ________________________________

At which organisation? ________________________________

From which sector?

◯ Ministry of Health - program on Neglected Tropical Diseases ◯ Ministry of Health - program on Snakebite Envenoming
◯ Ministry of Health - other
◯ Health Facility ◯ Research facility
◯ Patient organisation / community group
◯ other

Please describe your sector ________________________________

Could you give a short description of your activities in Snakebite Envenoming (1-2 sentences)? ____

Which department of the ministry is responsible for the Snakebite Envenoming policy? ________________________________

Is Snakebite Envenoming part of the National Neglected Tropical Diseases program?

- ◯ Yes
- ◯ No

How is Snakebite Envenoming embedded nationally? Can you please describe this? | ______________________________

Are there national policy documents on Snakebite Envenoming or national master plans on NTDs involving Snakebite Envenoming?
◯ Yes
◯ No

Can you upload these documents here one by one?

Do you have another document to upload?
◯ Yes
◯ No

Can you please upload the second document here?

Do you have another document to upload?
◯ Yes
◯ No

Can you please upload the third document here?

Are there regional policy documents on Snakebite Envenoming?
◯ Yes
◯ No

Can you upload these documents here one by one? Please upload all documents available to you.

Do you have another document to upload?
◯ Yes
◯ No

Can you please upload the second document here?

Do you have another document to upload?
◯ Yes
◯ No

Can you please upload the third document here?

Are there national or regional guidelines on the management of Snakebite Envenoming?
◯ Yes ◯ No

Can you upload these documents one by one? Please upload all documents available to you.

Do you have another document to upload?
◯ Yes
◯ No

Can you please upload the second document here?

Do you have another document to upload?
◯ Yes
◯ No

Can you please upload the third document here?

Who are the authors of these documents?	__________________________________
Do you use the WHO guidelines on the prevention and management of snakebites in Africa?	◯ Yes<br>◯ No
Are there patient organisations active in Snakebite Envenoming in your country? If not, are there patient organisations active in other NTDs in your country? Can you provide the names of these organisations?	__________________________________
Which barriers do you think play a role in the challenges in your country to generate evidence and translate this into policy in the field of Snakebite Envenoming?	__________________________________
Which barriers do you consider most relevant? Please select the most relevant barriers (max 5).	☐ Lack of relevant data and evidence<br>☐ Lack of access to relevant data and evidence<br>☐ Limited funding<br>☐ Snakebite envenoming not priority for policymakers or government<br>☐ Snakebite envenoming not priority for researchers<br>☐ Snakebite envenoming not priority for clinicians<br>☐ Limited time<br>☐ Lack of engagement with other stakeholders<br>☐ Unavailability of appropriate methodology or expertise<br>☐ Absence of knowledge translation platforms<br>☐ Limited availability of researchers<br>☐ Lack of national policy on snakebite envenoming<br>☐ Other,.........
Which other barrier?	__________________________________
Which factors in your country do you think facilitate the generation of evidence and the translation of this evidence into policy in the field of Snakebite Envenoming?	__________________________________
Which facilitators do you consider most relevant? Please select the most relevant facilitators (max 5).	☐ Motivation to contribute to public health<br>☐ Presence of interface for exchange<br>☐ Demand for scientific evidence<br>☐ Networking with experts<br>☐ Donors' funding and involvement<br>☐ Trust and respect of researchers<br>☐ Strong political will<br>☐ Prospects for career development<br>☐ Preparatory phase<br>☐ Clear and simple communication<br>☐ Other, ...
Which other facilitator?	____________________________

Which expertise or which contacts do you miss in your current position to generate evidence or to facilitate translation of this evidence into policy?

____

African Snakebite Research Hubs would aim to strenghten the evidence-base necessary to deliver snakebite treatments. In the hub, researchers can collaborate on public health research relevant for the region.

Prioritized research themes can be picked up by the Hub.

Which challenge or research topic would you consider to have the first priority and why?

____

Which three of the twelve potential topics below should be given the highest priority in African Snakebite Research Hubs?
(select max 3)

- ☐ Training of researchers
- ☐ Research on antivenom and new therapeutics
- ☐ Research on prevention of Snakebite Envenoming
- ☐ Research on improving health seeking behaviour
- ☐ Research on socio-cultural and economic risk factors
- ☐ Enhance disease burden monitoring and surveillance
- ☐ Build strong regional partnerships and alliances
- ☐ Facilitate research and policy development around healthcare cost mitigation
- ☐ Provide methodological support
- ☐ Engage with decision-makers to maximize uptake of research
- ☐ Support snakebite programs' integration into health systems
- ☐ Research on disability, mental health and stigma

Can you rank the three topics you selected? (first = highest priority)

	ranked as first	ranked as second	ranked as third
Training of researchers	◯	◯	◯
Research on antivenoms and new therapeutics	◯	◯	◯
Research on prevention of Snakebite Envenoming	◯	◯	◯
Research on improving health seeking behaviour	◯	◯	◯
Research on socio-cultural and economic risk factors	◯	◯	◯
Enhance disease burden monitoring and surveillance	◯	◯	◯
Build strong regional partnerships and alliances	◯	◯	◯
Facilitate research and policy development around healthcare cost mitigation	◯	◯	◯
Provide methodological support	◯	◯	◯
Engage with decision-makers to maximize uptake of research	◯	◯	◯
Support snakebite programs' integration into health system	◯	◯	◯
Research on disability, mental health and stigma	◯	◯	◯

How would the organisation you are representing be positioned with respect to African Snakebite Research Hubs, in terms of influence?

Influence in this regard can be described as the current capacity of your organisation to influence the initiative's impact. It includes the access, availability and mobilisation of resources; and/or the ability to mobilise other stakeholders and their resources; and/or the power to carry out potential activities or projects.

- ◯ Influence described as: None
- ◯ Influence described as: Low
- ◯ Influence described as: Medium
- ◯ Influence described as: High

What would be your organisation's attitude towards African Snakebite Research Hubs?

Attitude here means the current readiness of your organisation towards the initiative.

- ◯ Attitude: For
- ◯ Attitude: Neutral
- ◯ Attitude: Against

How would you describe your organisation's interest in the development of African Snakebite Research Hubs?

- ◯ Interest: None
- ◯ Interest: Low
- ◯ Interest: Medium
- ◯ Interest: High

To what extent do you think your organisation would like to be involved in the initiative?	◯ Would not like to be involved<br>◯ Only receiving information<br>◯ Be a consultative body<br>◯ Active participation in the process without making decisions<br>◯ Active participation in the process and in decision-making<br>◯ No answer/ Do not know
How could the organisation you are representing contribute to the aforementioned initiative?<br><br>Multiple answers allowed.	☐ planning the initiative<br>☐ participate in the organisational structure<br>☐ economic contribution/resources<br>☐ contribute to research initiatives<br>☐ provide methodological support<br>☐ developing and reviewing materials<br>☐ contribute to training initiatives<br>☐ contribute to team translating evidence to policy<br>☐ another:......
Which other contribution?	__________________________________
What do you think would be the biggest concern of your organisation in relation to an initiative of this kind?	____
Which three stakeholders do you consider national or international key stakeholders to involve in this survey on African Snakebite Research Hubs? Can you describe why you think these stakeholders should be involved?	____
Antivenoms are a crucial component of the treatment but availability is frequently challenging. Are there antivenom immunoglobins registered/licensed in your country?	◯ Yes<br>◯ No<br>◯ I don't know
Can you provide the names of the registered/licensed antivenoms and their manufacturers?	____
How do antivenom immunoglobulins get authorisation to be used in your country?	____
Do you think there is a role in the antivenom authorisation process which can be facilitated by African Snakebite Research Hubs?	◯ Yes<br>◯ No
What could African Snakebite Research Hubs do to assist in this process?	____
Would you be willing to answer additional questions in an in-depth interview on the potential role of African Snakebite Research Hubs?	◯ Yes<br>◯ No
Can you please provide your email address here?	__________________________________

Thank you very much for your participation!

If you have any additional comments, please write them in this text box. ________________________________
